# PD2/Paf1 depletion in pancreatic acinar cells promotes acinar-to-ductal metaplasia

**DOI:** 10.18632/oncotarget.2041

**Published:** 2014-05-30

**Authors:** Parama Dey, Satyanarayana Rachagani, Arokia P. Vaz, Moorthy P. Ponnusamy, Surinder K. Batra

**Affiliations:** ^1^ Department of Biochemistry and Molecular Biology, University of Nebraska Medical Center, Omaha, NE, U.S.A; ^2^ Fred & Pamela Buffet Cancer Center, Eppley Institute for Research in Cancer and Allied Diseases, University of Nebraska Medical Center, Omaha, NE, U.S.A; ^3^ Department of Pathology and Microbiology, University of Nebraska Medical Center, Omaha, NE, U.S.A

**Keywords:** ADM, cerulein, pancreatic cancer, PD2/Paf1

## Abstract

Pancreatic differentiation 2 (PD2), a PAF (RNA Polymerase II Associated Factor) complex subunit, is overexpressed in pancreatic cancer cells and has demonstrated potential oncogenic property. Here, we report that PD2/Paf1 expression was restricted to acinar cells in the normal murine pancreas, but its expression increased in the ductal cells of *Pdx1Cre; Kras^G12D^* (KC) mouse model of pancreatic cancer with increasing age, showing highest expression in neoplastic ductal cells of 50 weeks old mice. PD2/Paf1 was specifically expressed in amylase and CK19 double positive metaplastic ducts, representing intermediate structures during pancreatic acinar-to-ductal metaplasia (ADM). Similar PD2/Paf1 expression was observed in murine pancreas that exhibited ADM-like histology upon cerulein challenge. In normal mice, cerulein-mediated inflammation induced a decrease in PD2/Paf1 expression, which was later restored upon recovery of the pancreatic parenchyma. In KC mice, however, PD2/Paf1 mRNA level continued to decrease with progressive dysplasia and subsequent neoplastic transformation. Additionally, knockdown of PD2/Paf1 in pancreatic acinar cells resulted in the abrogation of Amylase, Elastase and Lipase (acinar marker) mRNA levels with simultaneous increase in CK19 and CAII (ductal marker) transcripts. In conclusion, our studies indicate loss of PD2/Paf1 expression during acinar transdifferentiation in pancreatic cancer initiation and PD2/Paf1 mediated regulation of lineage specific markers.

## INTRODUCTION

Pancreatic cancer (PC) is a lethal desmoplastic tumor with a 5-year survival rate of less than 6% [1]. It manifests cryptically with minimal early symptoms, and is compounded by a late clinical presentation and resistance to chemotherapy, which make this cancer a major challenge. Although much knowledge has been gained about the molecular mechanisms and genetic changes underlying pancreatic cancer pathogenesis, we have very little insight about the origin of this disease. Current tenet suggests ‘acinar-to-ductal metaplasia’ as the initiating event of pancreatic ductal adenocarcinoma. This phenomenon defined as the transformation of one differentiated cell type into another is characterized by loss of acinar cell polarity and gene signatures and gradual conversion of acinar structures into duct-like phenotype. During this process transitional metaplastic cells are formed, some of which ultimately produce the precursor PanIN lesions that predispose the pancreatic parenchyma to neoplastic transformation.

One of the earliest studies demonstrating the plasticity of acinar cells was done by Wagner and colleagues, who found that transgenic mice overexpressing TGFα develop extensive pancreatic fibrosis and detected trans-differentiation from acinar to ductal cells [2]. The trans-differentiation was accompanied by loss of acinar markers and gain in expression of ductal markers, and 50% of those mice developed dysplastic tumor lesions after 180 days. Subsequent studies revealed that these TGFα-induced changes in epithelial differentiation required downstream Notch signaling though nestin-positive intermediates [3]. Interestingly, when mutated *Kras*, a common presence in pancreatic cancer, was selectively expressed in acinar cells driven by the Elastase promoter, it induced acinar to ductal metaplasia [4]. Further studies demonstrated that Kras and Notch signaling cooperate to reprogram acinar cells towards a ductal morphology [5].The environmental cues that trigger acinar to ductal metaplasia are not well-understood; however, chronic inflammation does make the pancreatic environment prone to such metaplastic conversion [6], and this could explain the 16-fold increased risk of pancreatic adenocarcinoma in chronic pancreatitis patients [7-9]. A host of transcription factors take part in this transdifferentiation process, viz. Mist1, HNFα, Sox9, Isl1 and Gata6 [10-14]. Recently, Morris *et.al* suggested that β-catenin and a Kras-PKCι-MMP7 signaling axis works as a critical modulator of the Kras-dependent metaplastic reprogramming of acinar cells [15, 16].

PD2/Paf1 is a subunit of the RNA Polymerase II associated Factor (PAF) complex which is highly expressed in poorly differentiated PC. The PAF complex may play a critical role in cancer as other components of the PAF complex, viz. hCdc73, hLeo1, hCtr9 and hSki8, are aberrantly expressed in a number of cancers [17, 18]. PD2/Paf1, the main PAF subunit, is overexpressed in the poorly differentiated pancreatic cancer cell line Panc1 due to amplification of the 19q13 locus. Further studies revealed that ectopic expression of PD2/Paf1 in mouse fibroblast cell line induces neoplastic transformation with capacity to form tumors and metastatic deposits when implanted in athymic mice pancreas, indicating its oncogenic property[19]. Additionally studies from our laboratory demonstrated that PD2/Paf1 regulates the transcription of early endodermal lineage markers via its interaction with Oct3/4 protein to play a critical role in the maintenance of self-renewal and pluripotency of mouse embryonic stem cells [20]. In pancreatic cancer cells, PD2/Paf1, plays a role in cell cycle regulation by modulating expression of cyclins A1, A2, D1, E1, B1, and Cdk1 [21]. Since pancreatic cancer is widely associated with loss of cell cycle due to deregulated expression of cyclins and cdks (cyclin dependent kinases), hence we anticipate that aberrant PD2/Paf1 might have a role in pancreatic cancer pathogenesis. Epigenetic modifications and DNA template rearrangement can cause differential gene expression observed in PC. Recently, we demonstrated that PD2/Paf1 also regulates histone methylation and chromatin remodeling in pancreatic cancer cells [22].

Inspired by the expressional and functional significance of PD2/Paf1 in various pancreatic cancer cells [23, 24], we sought to determine the expression pattern of PD2/Paf1 in *Pdx1-Cre; Kras ^G12D^* (KC) spontaneous mouse model of PC. We found that in normal mice PD2/Paf1 is present only in pancreatic acini whereas its expression appears gradually in neoplastic ductal cells of KC mice with increasing age. PD2/Paf1 expression in KC mice was specific to the metaplastic ducts, where it was found to co-localize with the acinar and ductal cell markers. Further cerulein induced inflammation in normal mice vs. the KC mice showed differential expression of PD2/Paf1. Specifically, the inflammatory insult led to downregulation (which subsequently restored) of acinar PD2/Paf1 expression in normal mice, but in KC mice, it continued to be ablated with progressive neoplasia. In addition, we noted that in pancreatic acinar cells, Amylase and CK19 mRNA levels were altered by PD2/Paf1 downregulation, which suggested that PD2/Paf1 may be involved in the maintenance of the terminally differentiated acinar cell population, and thus, act as an impediment to *Kras^G12D^*-driven acinar cell de-differentiation and tumor initiation.

## RESULTS

### Expression of PD2/Paf1 in *Pdx1-Cre;Kras*^G12D^ murine pancreatic cancer model

**Figure 1:**
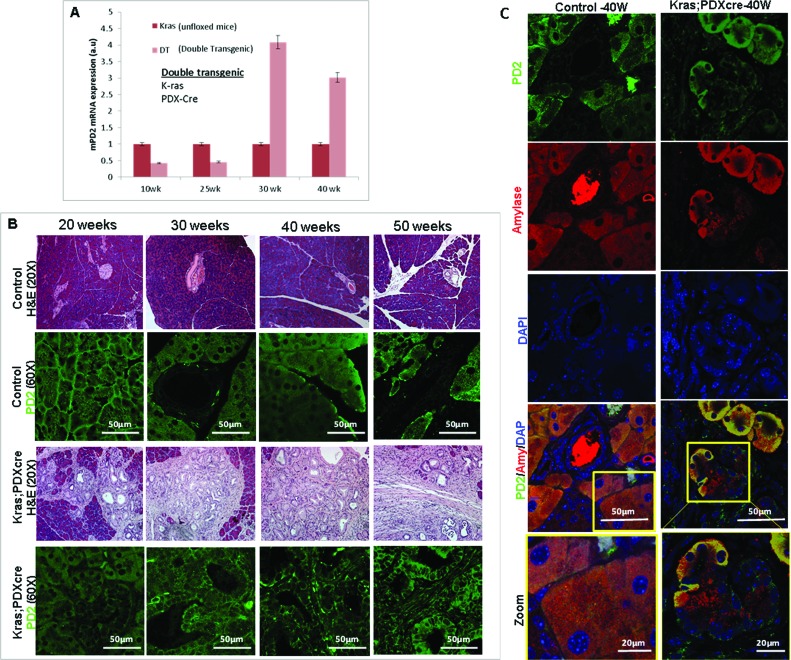
Expression of PD2/Paf1 in Pdx1-Cre;Kras^G12D^ murine pancreatic cancer model **(A)** Bar diagram representation of quantitative real time PCR analysis of PD2/Paf1 mRNA expression in various ages of mice from Pdx1-Cre;Kras^G12D^ (KC) pancreatic cancer progression model compared to corresponding age-matched normal mice. PD2/Paf1 mRNA expression is significantly (p<0.05) upregulated at 30 and 40 weeks of age in KC mice compared to healthy age-matched controls. **(B)** Immunofluorescence analysis of PD2/Paf1 in pancreatic tissues from normal wildtype (first and second row) versus KC mice (third and last row) at ages of 20, 30, 40 and 50 weeks. Normal mice showed acinar specific PD2/Paf1 expression but there was no ductal expression. On the other hand, PD2/Paf1 showed gradually increasing ductal expression with increasing age of mice with mutant Kras expression.[scale bar = 50μm] **(C)** Confocal images of PD2/Paf1 (green) expression in normal and KC mice along with acinar-cell specific marker amylase. In normal murine pancreas, only acinar cells show PD2/Paf1 expression while it appears in metaplastic ducts in pancreas of KC mice. (scale bar =50μm; zoom scale bar =20 μm)

We have followed the *Pdx1-Cre;Kras^G12D^* (KC) model to study the progression of pancreatic cancer [25]. The KC model serves as one of the most sophisticated of the currently established murine models for PC, emulating the human form of the disease. In this model, mutant *Kras^(G12D)^* signaling drives a series of molecular and histological changes leading to the development of ductal adenocarcinoma, recapitulating pancreatic cancer disease progression. Since PD2/Paf1 was differentially expressed in human pancreatic cancer cells, we explored its expression in the floxed *Kras^G12D^* animals (positive for both *Kras^G12D^* and *Pdx1-Cre*) along with their contemporary normal littermates harboring either *LSLKras^G12D^* or *Pdx1-Cre* at different time points during pancreatic cancer progression (N=8 for each time point). First, we analyzed by quantitative real time PCR, the mRNA expression of PD2/Paf1 in mice at different ages (in replicates). Comparison of the KC mice with age-matched littermate control mice led to the observation that the pancreatic expression of PD2/Paf1 mRNA at 10 and 20 weeks age appeared to have decreased in the *Kras* mice compared to the normal mice, even though that decrease did not reach statistical significance. There was, however, a significant increase in the pancreatic expression of PD2/Paf1 mRNA in the 30 and 40 week- old *Kras* mice compared to the control mice (p<0.05) (Figure 1A). Similar change in PD2/Paf1 expression was also observed in the KPC (*Kras^G12D^/p53/Pdx1-Cre*) mouse model of PC, where there was increased PD2/Paf1 mRNA level in 25-week old KPC mice, having advanced pancreatic ductal adenocarcinoma (Figure S1A).

Next, we investigated the expression of the PD2/Paf1 protein during pancreatic cancer progression by immunofluorescent staining of pancreatic tissues of the KC mice. Pancreatic tissues were harvested from 20, 30, 40 and 50-week old mice bearing *Kras^G12D^* mutation, and also from age-matched normal mice for examining PD2/Paf1 expression. In the normal mice, there was PD2/Paf1 expression in the acinar cells of pancreas (Figure 1B, top panel). The acinar specific PD2/Paf1 expression persisted in all age samples from the normal mice. However, in the KC mouse model, the PD2/Paf1 expression seemed to gradually appear in the ductal cells with increasing age of the mice. The ductal PD2/Paf1 expression pattern was most prominently visible in the dysplastic cancerous lesions of 40^th^ and 50^th^ week old-mice in the KC model (Figure 1B third and last panel). On the other hand, the acinar expression of PD2/Paf1 although present, appeared to decrease when compared to normal control tissues. Careful analysis of the tissues revealed that PD2/Paf1 expression in the ducts was restricted to certain specific ducts and not in all. Interestingly, these ductal structures were found to be metaplastic ducts representing intermediate structures with both acinar and ductal cell characteristics during the process of acinar-to-ductal metaplasia. We observed that PD2/Paf1 co-localized with amylase in such ‘pseudo-ductal’ structures (Figure 1C). A parallel observation was made in the KPC mouse model of PC, where PD2/Paf1 was absent from the ducts of normal mice, but appeared in the metaplastic and neoplastic ductal cells of KPC mice with increasing age (Figure S1B).

### Expressional variations of PD2/Paf1 during cerulein induced pancreatic injury in normal versus KC mice

**Figure 2:**
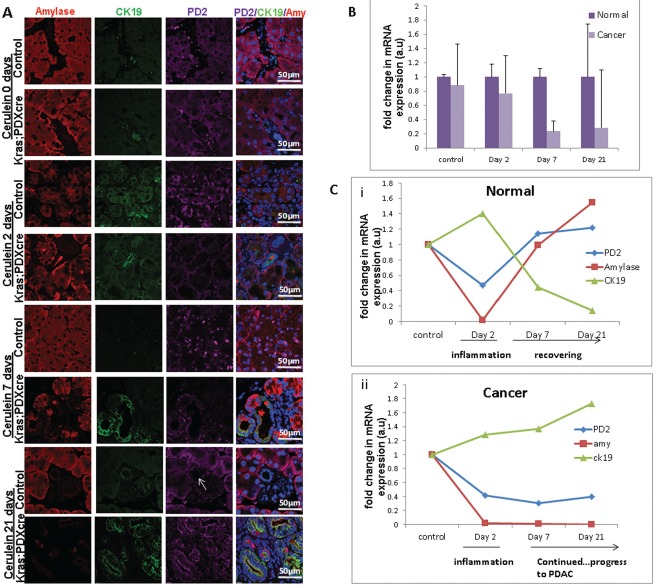
PD2/Paf1 expression during cerulein induced pancreatic injury in normal versus KC mice **(A)** Immunofluorescence images of PD2/Paf1 expression (purple) with acinar cell marker amylase (red) and ductal cell marker CK19 (green) in cerulein treated and saline treated mice at days 2, 7 and 21 post injection. Both normal and KC mice show acinar specific PD2/Paf1 expression under control conditions. At day 2 post injection, under cerulein induced metaplastic conversion, PD2/Paf1 expression appears in amylase and CK19 double positive metaplastic ducts in both normal and KC mice. However in normal mice at day 7 and 21, normal pancreatic parenchyma is restored with acinar cells showing robust amylase expression along with PD2/Paf1, which is further absent from the ducts (white arrow). In contrast, KC mice exhibited increasing dysplasia at days 7 and 21, with gradually increasing PD2/Paf1 expression in CK19 positive ducts. **(B)** Comparison of PD2/Paf1 mRNA levels between normal and KC mice under control and cerulein treated conditions showed decrease in PD2/Paf1 transcript levels in Kras^G12D^ mutant mice, with significant difference at day 7 and day 21 post trauma. **(C)** Real time PCR analysis of PD2/Paf1, amylase and CK19 mRNA levels in normal and KC mice for saline and cerulein treatment at various time points. (i) In normal mice PD2/Paf1 expression, along with amylase decreases due to inflammatory insult at day 2, but recovers by day 7 and 21. CK19 mRNA level, on the other hand, increases due to metaplastic changes in the pancreas at day 2, but falls back to basal level subsequently. (ii) In KC mice, PD2/Paf1 and amylase mRNA levels gradually decline with progressive dysplasia following cerulein treatment, along with a corresponding increase in CK19 mRNA level [scale bar =50μm]

Treatment with cerulein, a cholecystokinin analogue is known to aggravate pancreatic juice secretion with simultaneous inflammatory reaction. This inflammatory condition is associated with a subsequent injury to the pancreatic parenchyma along with widespread histological changes representing de-differentiation of acinar cells into ductal phenotype, more commonly known as acinar to ductal metaplasia. In our study, we injected cerulein to both normal and KC mice at 6 weeks of age, and studied the changes in PD2/Paf1 expression under inflammation induced ADM, from which the normal mice eventually recovered , but the mice containing mutant *Kras^G12D^* continued towards neoplastic progression (Figure S2). Metaplastic ducts characterized by the presence of both acinar marker amylase and ductal marker CK19 showed PD2/Paf1 expression (Figure 2A, Figure S3A), similar to our observation in KC mice progression model. In contrast, there was no PD2/Paf1 expression in the normal pancreatic ducts, which expressed only CK19 (Figure S3B). These trans-differentiating intermediate structures were visible 2 days after cerulein treatment in both normal and KC mice indicating inflammation induced metaplastic conversion. However, all normal mice examined either 7 days or 21 days after cerulein treatment, were found to have recovered completely with characteristic normal pancreatic parenchyma, consisting primarily of acinar cell population. But cerulein treatment to the *Kras^G12D^* mice showed progressively increasing dysplasia; there was widespread fibrosis in the day 7 mice and adenocarcinoma in the day 21 mice (Figure 2A,&E). Furthermore, examination of PD2/Paf1 expression in normal and KC mice (cerulein treated or untreated) showed abrogated PD2/Paf1 mRNA levels in KC mice with respect to normal mice at various time points (Figure 2B). In the normal mice, the PD2/Paf1 mRNA did decrease at day 2, in the presence of inflammation, but it gradually recovered to basal level as seen on days 7 and 21. A similar pattern is also observed in amylase mRNA level. In contrast, CK19 mRNA increased during the inflammatory insult and then decreased as normal pancreatic histology restored itself (Figure 2C i). However, in case of KC mice, both amylase and PD2/Paf1 mRNA level continued to decrease progressively with increasing dysplasia whereas the CK19 level remained high (Figure 2C ii).

### Cerulein treatment of pancreatic acinar cells

**Figure 3:**
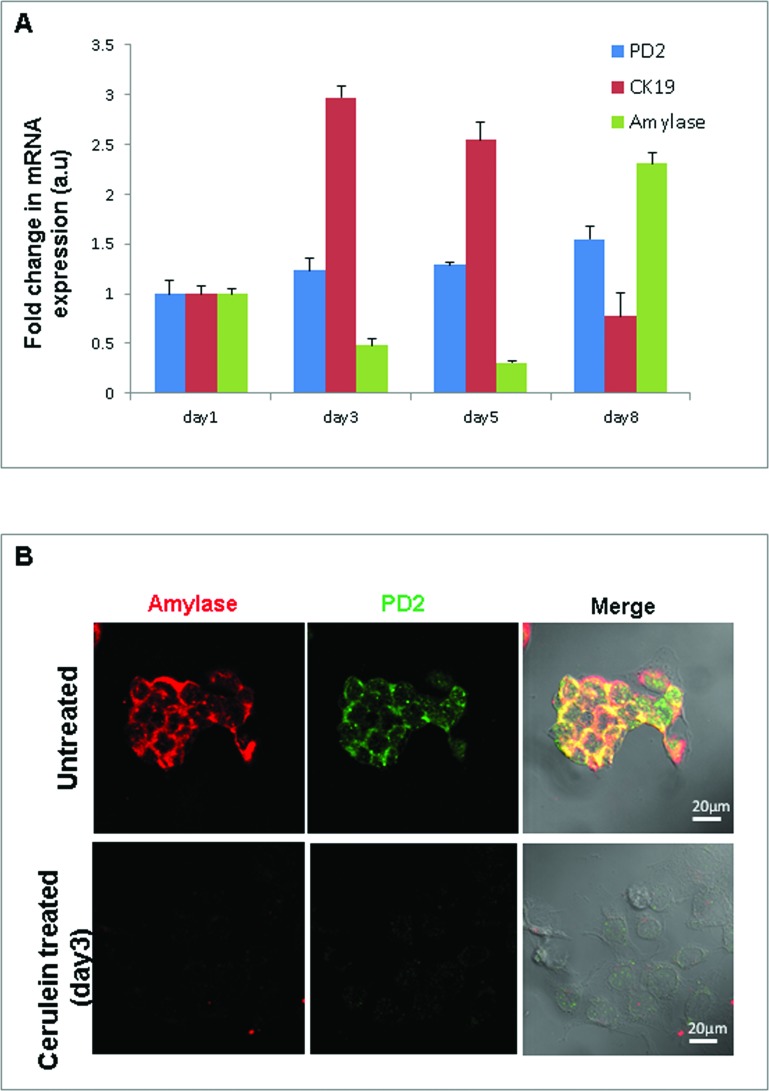
Cerulein treatment of pancreatic acinar cells **(A)** Real time PCR analysis of PD2/Paf1, amylase and CK19 mRNA levels in 266-6 pancreatic acinar cells in an *in vitro* model of cerulein induced acinar to ductal metaplasia. 266-6 cells upon cerulein treatment show gradually decreased expression of amylase (day 3, 5) with a corresponding increase in CK19 ductal marker expression. However, on treatment retrieval, amylase and CK19 expression is restored to basal levels (day8). PD2/Paf1 mRNA level does not show significant variation due to cerulein treatment in the 266-6 cells. **(B)** Immunofluorescence studies show decrease in PD2/Paf1 (green) and amylase (red) expression on day3 of cerulein treatment compared to untreated cells [scale bar =20 μm].

In order to elucidate the role of PD2/Paf1 in acinar to ductal metaplasia *in vitro*, we performed cerulein treatment in the 266-6 mouse pancreatic acinar cell line. These cells were treated with 10 nM of cerulein for 5 days and then allowed to recover for 3 days in 10% DMEM media. RNA isolated from the cells on days 1, 3, 5 and 8 after cerulein treatment were processed for Real-time PCR analysis of PD2/Paf1, amylase and CK19 genes. We observed that cerulein treatment led to a decrease in mRNA level of amylase with simultaneous increase in the level of CK19 gene. The levels of amylase and CK19 genes were restored back to normal on day 8. PD2/Paf1 expression also changed, although that did not reach statistical significance (Figure 3A). As control, NIH3T3 mouse fibroblast cells similarly treated with cerulein were used to examine whether these changes are specific to acinar cells. The untreated NIH3T3 cells did not show any amylase or CK19 expression, and cerulein treatment did not alter their expression either. On the other hand, a basal level of PD2/Paf1 expression was detectable in NIH3T3, which did not change due to cerulein treatment (Figure S4) indicating acinar cell specific modulation of PD2/Paf1 expression. Confocal microscopy showed that cerulein treatment of 266-6 cells led to reduced amylase and PD2/Paf1 expressions at day3 compared to untreated cells (Figure 3B), which indicated an acinar-ductal switch.

### Variation in acinar and ductal lineage markers due to knockdown of PD2/Paf1 in pancreatic acinar cells

**Figure 4:**
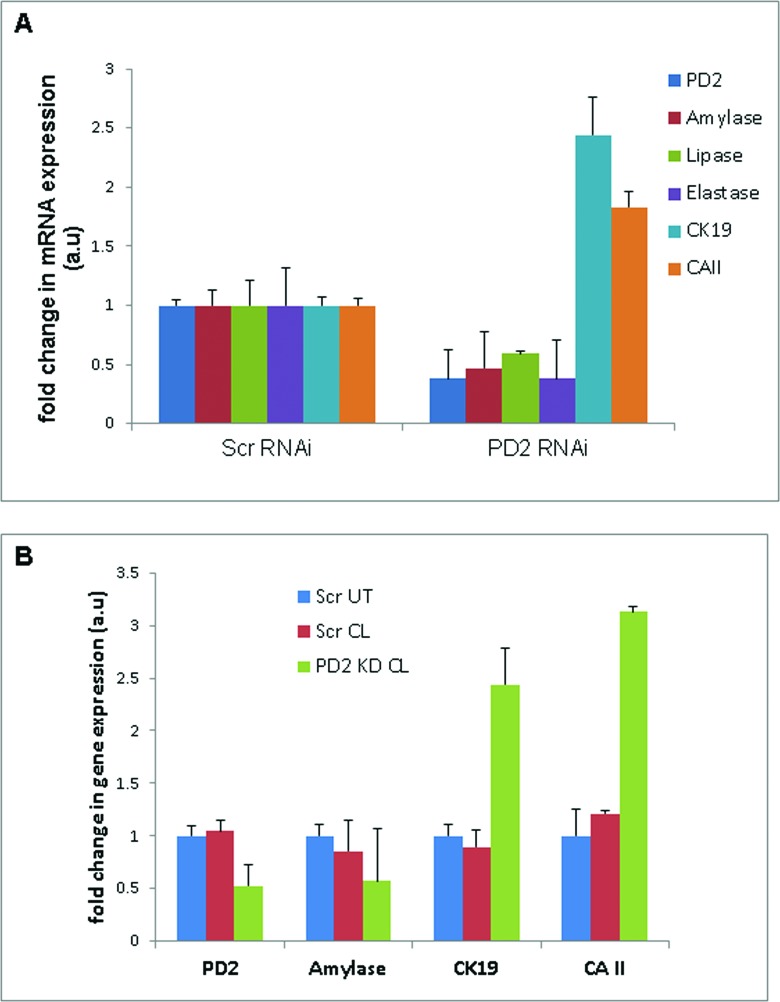
Variation in acinar and ductal lineage markers due to knockdown of PD2/Paf1 expression in pancreatic acinar cells (A) PD2/Paf1 was knocked down in the 266-6 pancreatic acinar cells using mouse PD2/Paf1 specific siRNA oligos. Analysis of mRNA expression of acinar (amylase, lipase, elastase) and ductal markers (CK19, CA II) in PD2/Paf1 KD 266-6 cells using Real-time PCR shows that decrease in PD2/Paf1 level led to abrogated expression of amylase, lipase and elastase with corresponding increase in CK19 and carbonic anhydrase (CA II) transcript levels. (B) Knockdown of PD2/Paf1 in 266-6 cells along with simultaneous cerulein treatment showed that in the absence of PD2/Paf1 there was further decrease in amylase expression due to cerulein treatment compared to scramble treated cells and a similarly robust increase in ductal marker (CK19, CA II) expression. Scr UT - Scrambled RNAi treated only, Scr CL- Scrambled RNAi + cerulein treatment, PD2KD CL- PD2 RNAi + cerulein treatment.

In order to explore whether PD2/Paf1 directly participates in the process of acinar to ductal trans-differentiation, we knocked down PD2/Paf1 in 266-6 mouse pancreatic acinar cells using specific siRNA oligos against mouse PD2/Paf1. PD2/Paf1 knockdown caused a decrease in amylase mRNA, and a significant increase in CK19 transcript (Figure 4A). This result correlated to what we have observed *in vivo*. We also investigated whether the downregulation of PD2/Paf1 would alter the expressions of other acinar and ductal lineage markers. PD2/Paf1 knockdown significantly downregulated two acinar cell markers such as enzymes like Elastase and Lipase mRNA expression. At the same time, ductal cell marker CA II (Carbonic anhydrase II) expression was enhanced by PD2/Paf1 knockdown compared to the control cells. In summary, these results indicated that loss in PD2/Paf1 expression results in abrogation of acinar-specific gene expression, and simultaneously, increases the expression of ductal lineage markers.

### Knockdown of PD2/Paf1 favors acinar cell plasticity

To delineate the role of PD2/Paf1 in pancreatic acinar-ductal transdifferentiation, we simultaneously treated the 266-6 cells with cerulein and PD2/Paf1 siRNA or scrambled control. 72 hours after the transfection and treatment, RNA was extracted from these cells. After 3 days of the treatments, the scrambled siRNA cells showed a slight increase in ductal gene expression (CK19 and CA II), but this increment was more than 2-fold higher in the PD2/Paf1 knockdown cells (Figure 4B). The acinar cell marker amylase, on the other hand, was downregulated upon cerulein treatment as was observed before, with further reduction in expression in PD2/Paf1-siRNA treated cells. These results indicate that loss of PD2/Paf1 expression in the acinar cells favors acinar cell plasticity and transdifferentiation into ductal morphology via differential expression of critical marker genes.

### PD2/Paf1 expression in ductal cells of acinar origin in pancreatic explant culture

**Figure 5:**
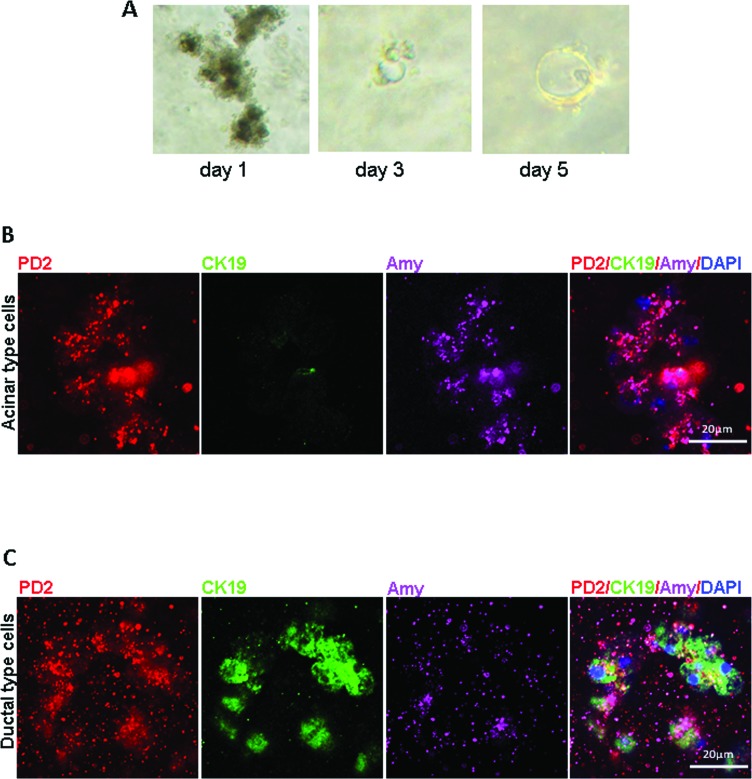
Expressional variation of PD2/Paf1 in pancreatic explant culture **(A)** Bright field pictures of pancreatic explant culture established from 6-week old Pdx1-Cre/Kras^G12D^ mouse at various time points. Day1 explant culture mainly represented acinar structures rich in dark zymogen granules that gradually transformed into smaller duct-like structures at day3 which further increased in size by day 5 of culture. **(B)** Confocal images showing PD2/Paf1(red) expression in amylase (violet) positive acinar structures, which were negative for CK19 ductal marker expression. **(C)** Immunofluorescence of PD2/Paf1 in CK19 positive (green) ductal cells arising from transdifferentiation of acinar cells.[scale bar = 20μm]

According to a previously proposed model, an explant culture of pancreatic acinar cells from 6 week-old KC mice was established using collagen [26]. We observed acinar cell clusters filled with dense zymogen granules on day 1, which gradually converted into small duct like structures at day 3, and went on to form even bigger structures at day 5 (Figure 5A). Immunofluorescence analysis of PD2/Paf1, amylase and CK19 expression revealed that PD2/Paf1 is expressed in the amylase positive acinar clusters, but they did not express CK19 (Figure 5B). As these mutants *Kras^G12D^* driven acinar cells spontaneously converted to a ductal morphology, we observed CK19 positive ductal cells that also expressed PD2/Paf1, even though at reduced levels compared to that in the acinar cells (Figure 5C). Thus, PD2/Paf1 was present both in acinar cells and in ductal cells of acinar origin in the explant culture.

## DISCUSSION

Metaplasia, the transformation of one tissue or cell type into another, is a physiological process that produces atypical or dysplastic terminal cells, and is relevant for development as well as for pathological conditions. The stimulus for such dramatic trans-differentiation may be an inflammatory insult or an injury, and both can ultimately lead to neoplastic transformation. Such appears to be the case of pancreatic cancer, in which there is a random acinar-ductal conversion in the pancreas. This acinar to ductal metaplasia event is held as the current model for the origin of pancreatic ‘ductal’ adenocarcinoma. Recent studies have implicated the role of various transcription factors and signaling pathways in mediating this process. The PD2/Paf1 subunit of the PAF complex, through its association with RNA Pol II plays an important role in transcription elongation and mRNA processing [27]. It is also known to co-transcriptionally coordinate events such as histone modification and chromatin remodeling [28, 29]. In this study we demonstrate for the first time a unique role of this novel protein PD2/Paf1 in pancreatic acinar to ductal metaplasia during murine pancreatic cancer progression.

Previous studies have shown that aberrant expression of different subunits of the human PAF complex is associated with tumorigenesis. The PD2/Paf1 subunit which constitutes the core of this complex, merits particular attention as it is overexpressed in poorly-differentiated pancreatic cancer cell lines as well as in mouse xenografts. It is known to be a cell cycle regulatory molecule, and also known to be involved in the maintenance of stem cell self-renewal and pluripotency. Our recent study revealed that PD2/Paf1 plays an important role in the maintenance of self-renewal and drug resistance of pancreatic cancer stem cells [30]. Our current study revealed that PD2/Paf1 is expressed only in normal pancreatic acinar cells, but not in the ducts. In murine pancreatic cancer progression models, however, PD2/Paf1 accumulated in the neoplastic ductal cells. Considering the role of PD2/Paf1 in stem cell differentiation, it is plausible that in the normal situation, its acinar-restricted expression points to a possible role of PD2/Paf1 in maintaining the differentiated state of acinar cells. Our observation that PD2/Paf1 is expressed in the metaplastic ducts implies its possible involvement in transdifferentiation. The metaplastic ducts are intermediate structures in the process of acinar to ductal metaplasia, which histologically look like acinar structures with enlarged hollow lumen and are characterized by presence of both the acinar marker Amylase, and the ductal marker CK19. We observed that PD2/Paf1 co-localized with such dual positive trans-differentiating tubules.

Pancreatitis is one of the major risk factors for pancreatic cancer. It can be induced in mice by cerulein injection, which induces histological changes seen in acinar to ductal transdifferentiation. We found that cerulein-induced inflammation causes PD2/Paf1 to localize in the metaplastic ducts. Interestingly mouse genotype determined the response to cerulein inflammation. Normal mice recovered from cerulein injury after 2 days, but mice with mutant *Kras^G12D^* developed greater dysplasia that more rapidly progressed to adenocarcinoma. PD2/Paf1 expression responses to the inflammatory insult were also dependent on the mice genotype. In KC mice, upon cerulein-induced inflammation, PD2/Paf1 mRNA level continuously decreased in tandem with amylase. This was in contrast to the restoration of PD2/Paf1 expression in normal mice during the recovery phase. In mouse embryonic stem cells, loss of PD2/Paf1 expression has been linked to increased differentiation potential towards an endodermal lineage [20]. Since in normal mice, PD2/Paf1 is restricted only to the acinar cells, it is plausible that PD2/Paf1 has a similar role in the maintenance of acinar cell differentiated state. It appears that in normal animals, PD2/Paf1 helps in the recovery and restoration of the pancreatic parenchyma post-trauma, but in presence of mutant *Kras^G12D^* oncogene, inflammation-ablated PD2/Paf1 expression cannot be regained, which allows acinar cells to be reprogrammed into a ductal phenotype, predisposing them towards neoplastic transformation.

A recent study demonstrated that the loss of EZH2, which is a part of polycomb repressor complex that catalyzes histone H3K27 methyltransferase, results in impaired pancreatic regeneration and accelerated *KRas^G12D^*-driven neoplasia [31]. That study is the first demonstration that in addition to tissue differentiation, the epigenetic modifier EZH2 can also play a role in tissue repair by promoting the regenerative proliferation of progenitor cells, thereby preventing tumorigenesis. Loss of PD2/Paf1 has been shown to promote differentiation of mouse embryonic stem cells towards endodermal lineage through changes in Gata4, Gata6 and Fgf8 gene expression [20]. Pancreas specific deletion of Gata6 leads to increased acinar apoptosis and proliferation, acinar-to-ductal metaplasia and adipocyte trans-differentiation through transcriptional regulation of digestive enzymes [10]. Therefore, we speculate that downregulation of PD2/Paf1 may also be responsible for the repair and re-establishment of the acinar cell population following injury. However, the presence of *Kras* (G12D) mutation makes the pancreatic acinar cells refractory to PD2/Paf1-mediated recovery. Since PD2/Paf1 has been shown to regulate H3K4 methylation status in pancreatic cancer cells [22], this function might account for the role of PD2/Paf1 in maintenance of acinar cell lineage.

Our *in vivo* observations were further supported by the *in vitro* studies with pancreatic acinar cells. The variation in expression of acinar genes such as Elastase and Amylase upon PD2/Paf1 downregulation in pancreatic acinar cells supports our hypothesis of PD2/Paf1-mediated regulation of acinar differentiation. Downregulation of acinar transcription factors such as Mist1 or upregulation of ductal transcription promoters such as HNF6 and Sox9 have been associated with promotion of acinar to ductal metaplasia [12, 13]. Our results suggest that PD2/Paf1 might also play a similar role in the induction of acinar cell reprogramming by regulating acinar gene expression. Further our observations from simultaneous PD2/Paf1 knockdown and cerulein treatment experiments in pancreatic acinar cells suggest that the ablation of PD2/Paf1 expression is not necessary but is favorable for acinar to ductal trans-differentiation.

Pancreatic explant culture of acinar cells derived from the KC mice provided us with an excellent *in vitro* model to study spontaneous transdifferentiation of cells, including the acinar to ductal metaplasia. We observed that PD2/Paf1 was expressed in acinar cells as well as in acinar cell-derived ductal cells in explant culture; and these results support our *in vivo* observation. PD2/Paf1 expression is restricted to acinar cells and its expression is ablated during acinar transdifferentiation, possibly contributing towards CK19 upregulation. PD2/Paf1 expression appears to be retained at a low level in the ductal cells of acinar origin and subsequently increases during the later stages of pancreatic cancer progression. Interestingly, the appearance of PD2/Paf1 expression in neoplastic ductal cells as opposed to its absence from the normal ducts suggests that the PD2/Paf1 positive ducts might have an acinar lineage. Further lineage tracing experiments *in vivo* will be required to explore this hypothesis. The elevated expression of PD2/Paf1 in pancreatic cancer may suggest that it plays a biphasic role during pancreatic cancer progression; its initial downregulation allows acinar cell de-differentiation and in later stages its elevated expression favors pancreatic tumor progression.

**Figure 6:**
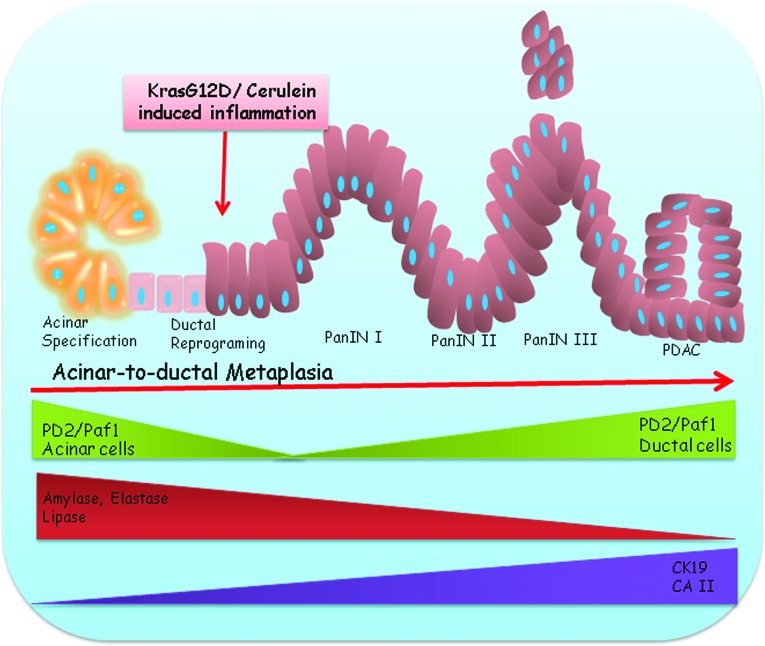
Involvement of PD2/Paf1 in pancreatic acinar-to-ductal metaplasia Presence of mutated Kras (G12D) or cerulein mediated inflammatory insult is known to induce transdifferentiation of pancreatic acinar cells into a ductal phenotype. PD2/Paf1, originally restricted to pancreatic acinar cells only, is ablated during this metaplastic conversion to facilitate downregulation of acinar cell marker genes (Amylase, Elastase, Lipase) with simultaneous upregulation of ductal markers (CK19,CA II). However, the PD2/Paf1 level increases with increasing dysplasia and eventually in neoplastic ductal cells with progressive appearance of preneoplastic PanIN lesions in the mutnat Kras mice. Therefore, PD2/Paf1 has a bi-phasic role during pathogenesis of pancreatic cancer, wherein its initial downregulation favors acinar to ductal metaplasia while elevated expression in later stages assists in disease progression.

In summary, our results show that PD2/Paf1 expression is restricted to the pancreatic acinar compartment under non-pathological conditions (Figure 6). However, during inflammation or neoplastic transformation, PD2/Paf1 expression is downregulated in the acinar cells with simultaneous abrogation of acinar cell markers, such as amylase, elastase and lipase, and upregulation of ductal markers, such as CK19 and CAII (Figure 6). This might facilitate gradual reprogramming of acinar cells into ductal cells and subsequently their neoplastic transformation. Since PD2/Paf1 high expression is seen specifically in the metaplasia-derived ductal cells, it might be a possible marker of their acinar origin.

## MATERIALS AND METHODS

### Cell Lines and Tissue Culture

Murine pancreatic acinar cell line 266-6 (CRL-2151™) was obtained from ATCC. The cells were cultured in DMEM media (Sigma) supplemented with 10% fetal bovine serum (Sigma) and 1% pencillin-streptamycin (100 units/ml) solution (Sigma) at 37°C and 5% CO_2_. Culture medium was changed every 2 days and cells were subcultured by trypsin-EDTA treatment.

### Mouse lines

The B6.129-*Kras^tm4Tyj^* (01XJ6) and B6. FVB-Tg (Ipf1-cre)1Tuv (01XL5) mice were obtained from the NCI Mouse Models of Human Cancers Consortium (MMHCC) (Frederick, MD, USA). These animals (LSL-*Kras^G12D^* and *Pdx1-Cre*) were crossed to remove the LSL cassette in order to activate *Kras^G12D^* (*Pdx1-Cre*;floxed *Kras^G12D^*) allele in the pancreas of the mouse. The F1 progeny was genotyped for *Kras* as well as *Pdx1-Cre* by using specific primers for *Kras* and *Pdx1-Cre* by Polymerase chain reaction (PCR). Animals that were positive for *Kras^G12D^* and *Pdx1-Cre* expressed the mutated *Kras^G12D^* allele in the pancreas. The floxed *Kras^G12D^* animals (positive for both *Kras* and *Pdx1-Cre*) and their contemporary littermates positive for either *LSLKras^G12D^* or *Pdx1-Cre* were euthanized at 7, 10, 25, 30, 40 and 50 weeks of age (eight animals/group/time points). Animals were maintained by providing food and water ad libitum and also kept in a 12h dark/light condition. We have followed the U.S. Public Health Service guidelines for the care and use of laboratory animals with the approval of UNMC-IACUC.

### RNA Interference and transient transfection

Mouse PD2/Paf1 was targeted with specific siRNA (sequence 5'- CGAGTCAAGTACTGCAATA-3)'. Synthetic sense and antisense oligonucleotides (Dharmacon, Lafayette, CO) were annealed in 100 mM potassium acetate, 30 mM Hepes-KOH (pH 7.4), and 2 mM magnesium acetate for one minute at 90°C and one hour at 37°C, and frozen. For transient transfection, the 266-6 cells were seeded at 70% confluency 24hrs prior to transfection. Following serum starvation for four hours, the cells were transfected using Lipofectamine 2000 reagent (Invitrogen) using manufacturer's protocol. Four hours following transfection, the cells were replenished with serum containing media and protein and RNA were isolated from the transfected cells at 48 and 72 hours post-transfection.

### RNA Isolation and QRT-PCR

Total RNA was collected from whole mouse pancreatic tissue after tissue homogenization, using mirVana RNA isolation kit (Invitrogen). Total cellular RNA was extracted from 266-6 cells using the RNAeasy kit (Qiagen) and processed for reverse transcription. After reverse-transcription, mRNA levels of different genes were assayed by quantitative real-time PCR using SYBR Green incorporation. The expression of all genes was normalized to that of the internal control β-actin and expressed relative to the indicated reference sample (average ± S.D. of triplicate reactions). The expressions of different acinar and ductal marker genes were compared between the scrambled and PD2/Paf1 knockdown 266-6 cells by the two-tailed Student's t-test. A p-value of <0.05 was considered statistically significant. Primer sequences for Real-time PCR are provided in the table S1.

### Immunofluorescence in mice tissues

The mice tissue slides were baked overnight at 58°C and then dewaxed by washing in xylene solution three times for 10 mins each. The slides were then rehydrated in decreasing grades of alcohol (100% - 20%) and finally washed in 1X PBS. Next, the tissues were fixed in 100% methanol for 1 hour followed by antigen recovery by heating in 0.01M citrate buffer in a microwave for 15mins. The tissues were then blocked with 10% normal goat serum and incubated with the primary antibodies - mouse anti-PD2/Paf1 1:100], rabbit anti-amylase [Sigma, 1:300] and rat anti-CK19 [TROMA III, 1:200] overnight at 4°C. PD2/Paf1 monoclonal antibody was raised in our lab corresponding to amino acids 327–348 peptide which is exactly humongous to mouse PD2/Paf1 molecule. This antibody has been used for the mouse ESC lines and NIH3T3 lines [19, 20]. After overnight incubation, the slides were washed with 1X PBS three times, incubated with secondary antibody (FITC conjugated anti-rat, Texas Red conjugated anti-rabbit and FITC conjugated anti-mouse) for 30mins and then washed again in PBS. The slides were mounted by DAPI containing Vectashield mounting solution. The slides were analyzed using Zeiss (Carl Zeiss Microimaging, Thornwood, NY) confocal laser-scanning microscope, and representative photographs were captured digitally using the 710 LSM software.

### Cerulein treatment *in vitro* and *in vivo*

Mice were treated with the cholecystokinin analogue Cerulein to induce pancreatitis following the staggered protocol as described before [32]. Briefly, the mice were given 8 hourly intraperitoneal injections with cerulein (75μg/kg body weight) on two alternate days. Counting the last day of treatment as day 0, the mice were sacrificed at 2, 7 and 21 days post-treatment and tissues were collected for mRNA, protein and immunohistochemical analysis. For cerulein treatment *in vitro*, 266-6 cells were treated with 100nM of cerulein for 5 days and then allowed to recover in 10% DMEM media for 3 days. RNA was collected on day 1, 3, 5 and 8 and processed for real-time PCR analysis.

### Explant Culture

Explant culture of pancreatic acinar cells from 6 week old *Kras^G12D;^Pdx1-Cre* (KC) mice was established using a previously published protocol [33, 34]. Briefly, 24-well plate was pre-coated with neutralized rat tail collagen (RTC) and allowed to solidify at 37°C. 6 week-old KC mice were sacrificed and pancreas was resected out, washed in cold HBSS and subjected to digestion in 0.2mg/ml Collagenase P solution for 10 minutes at 37°C. The reaction was stopped by adding cold HBSS containing 5% FBS and centrifuged at 720g for 2 mins at 4°C. The pellet was resuspended in 10ml of 5% FBS containing HBSS and allowed to pass through 500μm and 105μm sterile mesh consecutively. The final filtered solution was added to the top of a 30%FBS cushion and centrifuged at 180g for 2 mins at 4°C. The pellet was further resuspended in 10 ml of cold RPMI1640 media supplemented with penicillin G (1000 U/ml), streptomycin (100 μg/ml), 10%FBS solution, 100ug/ml Soybean Trypsin Inhibitor and 1ug/ml dexamethasone. It was then mixed with equal volume of neutralized RTC and added to the collagen embedded 24-well plate. After solidification, 500μl RPMI media was added to each well.

## SUPPLEMENTARY FIGURES AND TABLE


