# Supplementary material for: PD2/Paf1 depletion in pancreatic acinar cells promotes acinar-to-ductal metaplasia
Source: Oncotarget. 2014 May 30;5(12):4480–91. doi: 10.18632/oncotarget.2041 (PMC4147339; doi:10.18632/oncotarget.2041)
Supplement: Supplementary file 1 [file oncotarget-05-4480-s001.pdf]

PD2/Paf1 depletion in pancreatic acinar cells promotes acinar-to-ductal metaplasia

Supplementary Material

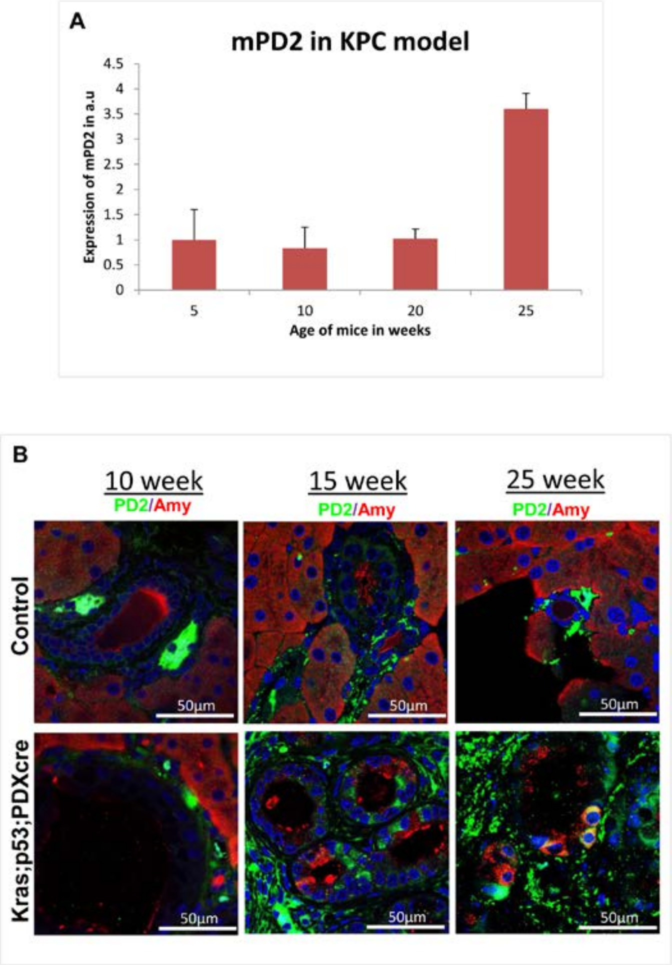

Figure S1: Expression of PD2/Paf1 in KPC mouse model of pancreatic cancer. (A) mRNA expression of PD2/Paf1 in different ages of mice from the Kras^{G12D}/P53/Pdx1-Cre (KPC) mouse model of pancreatic cancer. There is a significant increase in the mRNA level of PD2/Paf1 in 25 week old mice (having pancreatic ductal adenocarcinoma) compared to that of a 5 week old mice (normal pancreas). **(B)** Confocal analysis of PD2/Paf1 expression in KPC mice model of different ages. Pancreas from normal mice shows acinar specific PD2/Paf1 expression throughout all age groups (upper panel). In KPC mouse model, there is progressive increase in PD2/Paf1 expression in the neoplastic ductal cells with increasing age of mice (lower panel).[scale bar =50μm]

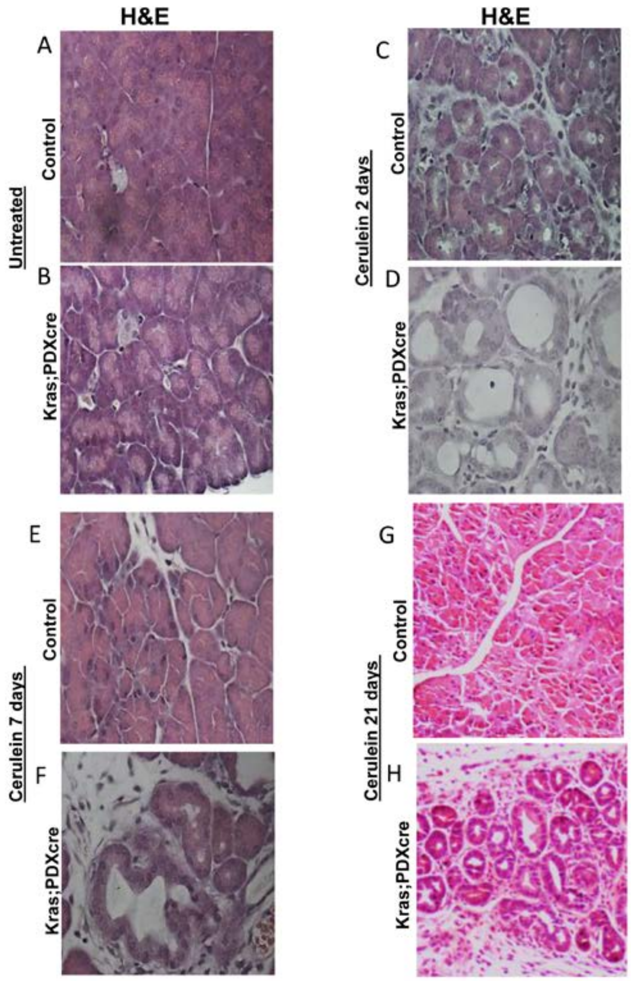

Figure S2: Histological analysis of normal and KC mice with and without cerulein treatment. Hematoxylin staining of pancreatic lesions from normal and $Kras^{G12D}$/Pdx1-Cre (KC) mice with and without cerulein treatment. Untreated normal (A) and KC mice (B) showed normal pancreatic parenchyma consisting mostly of acinar structures. After 2 days of cerulein treatment, both normal (C) and KC mice (D) exhibited widespread metaplasia, with acinar clusters having enlarged lumen and partial ductal characteristics. The normal mice recover from cerulein induced inflammatory insult by days 7 (E) and 21 (G) showing normal pancreatic parenchyma. However in KC mice, there is progressive dysplasia and increased appearance of ductal structures at days 7 and 21 (F, H).

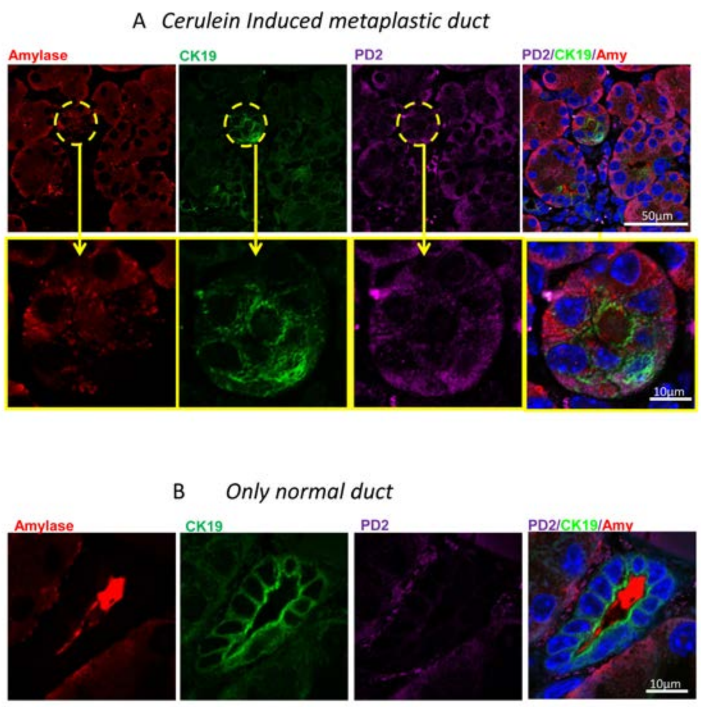

Figure S3: PD2/Paf1 expression in normal vs metaplastic ducts. (A) PD2/Paf1 (violet) expression in metaplastic ducts shows its localization in the acinar-to-ductal transdifferentiating structures which show both amylase (red) and CK19 (green) expression [scale bar 50 µm]. The lower panel is the magnified version of the upper panel showing the metaplastic duct [scale bar = 10µm]. **(B)** The normal murine pancreas shows no PD2/Paf1 expression in the ducts, which express CK19 but not amylase, but is expressed by the surrounding acinar cells. [scale bar = 10µm]

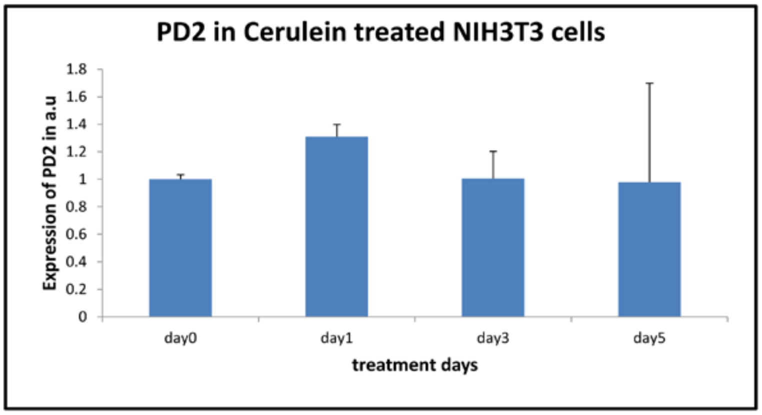

Figure S4: Expression of mouse PD2/Paf1 expression in cerulein treated NIH3T3 cell. Quantitative Real-time PCR analysis of PD2/Paf1 expression in NIH3T3 mouse fibroblast cells treated with 10nM of cerulein over a period of 5 days. There was no significant change in mouse PD2/Paf1 expression in the NIH3T3 cells with cerulein treatment at different days.

Supplementary table 1:

Gene	Primer Sequence
Mouse amylase	FP – 5’-CAAAATGGTTCTCCCAAGGA-3’<br>RP – 5’-ACATCTTCTCGCCATTCCAC-3’
Mouse cytokeratin 19 (CK19)	FP – 5’-ACCCTCCCGAGATTACAACC-3’<br>RP- 5’-CAAGGCGTGTTCTGTCTCAA-3’
Mouse Elastase	FP- 5'-ACTATGTCCAGCTGGGTGTTC-3',<br>RP- 5'-CAGTAAGAGGAGCTGGAGCAG-3'
Mouse Lipase	FP- 5'-GGCATTGTTGGTTAACGTTCT-3',<br>RP- 5'-AATTGCGTCCACAAACTGAG-3'
Mouse Carbonic Anhydrase (CA II)	FP-5'-TGATGACTCTCAGGACAATGC-3',<br>RP-5'-TGTTCCAGTGAACCAAGTGAA-3’
Mouse Paf1	FP-5’-CCAGTTGGAAAACCACGAAC-3’<br>RP-5’-CTCGCTGCCTTCTTTCTCAC-3’
